# Gene expression profiling of hepatocarcinogenesis in a mouse model of chronic hepatitis B

**DOI:** 10.1371/journal.pone.0185442

**Published:** 2017-10-02

**Authors:** Takuto Nosaka, Tatsushi Naito, Katsushi Hiramatsu, Masahiro Ohtani, Tomoyuki Nemoto, Hiroyuki Marusawa, Ning Ma, Yusuke Hiraku, Shosuke Kawanishi, Taro Yamashita, Shuichi Kaneko, Yasunari Nakamoto

**Affiliations:** 1 Second Department of Internal Medicine, Faculty of Medical Sciences, University of Fukui, Yoshida-gun, Fukui, Japan; 2 Department of Gastroenterology and Hepatology, Graduate School of Medicine, Kyoto University, Sakyo-Ku, Kyoto, Japan; 3 Faculty of Nursing Science, Suzuka University of Medical Science, Suzuka, Mie, Japan; 4 Department of Environmental and Molecular Medicine, Mie University Graduate School of Medicine, Tsu, Mie, Japan; 5 Faculty of Pharmaceutical Sciences, Suzuka University of Medical Science, Suzuka, Mie, Japan; 6 Department of Gastroenterology, Kanazawa University Graduate School of Medical Science, Kanazawa, Ishikawa, Japan; University of Navarra School of Medicine and Center for Applied Medical Research (CIMA), SPAIN

## Abstract

**Background:**

Hepatocellular carcinoma (HCC) is a common complication of chronic viral hepatitis. In support of this notion, we have reported that hepatitis B surface antigen (HBsAg)-specific CD8^+^ T lymphocytes critically contribute to inducing chronic liver cell injury that exerts high carcinogenic potential in a hepatitis B virus (HBV) transgenic mouse model. The dynamics of the molecular signatures responsible for hepatocellular carcinogenesis are not fully understood. The current study was designed to determine the serial changes in gene expression profiles in a model of chronic immune-mediated hepatitis.

**Methods:**

Three-month-old HBV transgenic mice were immunologically reconstituted with bone marrow cells and splenocytes from syngeneic nontransgenic donors. Liver tissues were obtained every three months until 18 months at which time all mice developed multiple liver tumors. Nitrative DNA lesions and hepatocyte turnover were assessed immunohistochemically. Gene expression profiles were generated by extracting total RNA from the tissues and analyzing by microarray.

**Results:**

The nitrative DNA lesions and the regenerative proliferation of hepatocytes were increased during the progression of chronic liver disease. In a gene expression profile analysis of liver samples, the chemokine- and T cell receptor (TCR)-mediated pathways were enhanced during chronic hepatitis, and the EGF- and VEGF-mediated pathways were induced in HCC. Among these molecules, the protein levels of STAT3 were greatly enhanced in all hepatocyte nuclei and further elevated in the cytoplasm in HCC tissue samples at 18 months, and the levels of phosphorylated TP53 (p-p53-Ser 6 and -Ser 15) were increased in liver tissues.

**Conclusions:**

HBV-specific immune responses caused unique molecular signatures in the liver tissues of chronic hepatitis and triggered subsequent carcinogenic gene expression profiles in a mouse model. The results suggest a plausible molecular basis responsible for HBV-induced immune pathogenesis of HCC.

## Introduction

The development of hepatocellular carcinoma (HCC) is a complex multifactorial process in which many years of chronic hepatitis plays a major role.[1, 2] In patients with chronic hepatitis B and C, the virus-specific CD4^+^ and CD8^+^ T lymphocytes have been reported to play a role in the immunopathogenesis of liver disease.[3–6] In transgenic mouse models of hepatitis B virus (HBV), transfer of CD4^+^ and CD8^+^ T cell clones specific for the viral antigens induced acute liver cell injury.[7–9] In an effort to evaluate the carcinogenic potential of chronic inflammation, we have established a model of prolonged immune-mediated hepatitis using HBV transgenic mice that express the viral envelope proteins in their hepatocytes.[10]

Furthermore, the lymphocyte subset transfer revealed that the pathogenetic events caused by CD8^+^ cytotoxic T lymphocytes (CTLs) are primarily responsible for the development of chronic liver disease that enhances tumor incidence.[11]

CD8^+^ CTL-induced continuous inflammation is sufficient to trigger the process of hepatocarcinogenesis in the absence of preexisting viral transactivation or genetic changes in chronic HBV infection.[10, 12, 13] During the process of cancer development, molecular events may accumulate in intracellular and extracellular microenvironments to establish the pathological signatures of tumor tissues distinct from non-cancer conditions. In the current study, molecular events specific for long-term persistent inflammation and tumor tissues were detected in a model of chronic hepatitis B in which the virus-specific CD8^+^ CTLs establish a cycle of hepatocyte death and regeneration. Some the genes were activated in the process of chronic hepatitis, while other genes were exclusively changed in tumor tissues. The results demonstrate that HBV-specific immune responses caused unique molecular signatures in the liver tissues and triggered subsequent procarcinogenic processes in the hepatocytes.

## Materials and methods

### HBV transgenic mice

Hepatitis B surface antigen (HBsAg) transgenic mouse lineage 107-5D (official designation Tg[Alb-1,HBV]Bri66) (inbred B10D2, H-2d) was kindly provided by Dr. F.V. Chisari (The Scripps Research Institute, La Jolla, California)[14]. The 107-5D lineage contains the entire HBV envelope-coding region (subtype ayw) under the constitutive transcriptional control of the mouse albumin promoter[14]. These mice express the HBV small, middle and large envelope proteins in their hepatocytes[14]. They are immunologically tolerant to HBsAg at the T cell level[15] and they display no evidence of liver disease during their lifetime, without the adoptive transfer of HBsAg-specific CTLs[7, 8, 14]. There is no X-RNA or X-protein expression detectable in the livers of these animals (unpublished observations).

### Ethics statement

All the animal experiments were satisfied according to the Guidelines for the Care and Use of Laboratory Animals in Takara-machi Campus of Kanazawa University, and the Regulations for Animal Research at University of Fukui and were approved by the ethical committee of the animal experiments of Kanazawa University and University of Fukui. All surgery was performed under sodium pentobarbital anesthesia, and all efforts were made to minimize suffering.

### Disease model

The animal model of chronic hepatitis was generated as described previously[10]. Briefly, 3-month-old male HBsAg transgenic mice were thymectomized, irradiated (900 cGy), and their hemopoietic system was reconstituted with bone marrow cells from syngeneic nontransgenic B10D2 (H-2d) mice. One week after bone marrow transfer, the animals received 2 x 10^8^ splenocytes from nontransgenic B10D2 (H-2d) mice that had been infected intraperitoneally with a recombinant vaccinia virus expressing HBsAg (HBs-vac) 3 weeks before the splenocyte transfer[7]. The resultant hepatocellular injury was monitored biochemically by measuring serum alanine aminotransferase (ALT) activity[16]. Results were expressed as mean units per liter ± SD of serum ALT (sALT) activity. Tumor development was assessed by abdominal palpation and confirmed by autopsy. Tissue samples were fixed in 10% zinc-buffered formalin (Anatech Ltd., Battle Creek, MI), embedded in paraffin, sectioned (3 μm) for immunohistochemical analysis.[16]. Liver tissue was also snap-frozen in liquid nitrogen and stored at—80°C for molecular analysis.

### cDNA microarray

Gene expression microarray analysis was performed using the materials and protocols from the Agilent Technologies two-color gene expression platform [Agilent Mouse 44k (012694_D)]. Briefly, total RNA was isolated from snap-frozen hepatic parenchyma and HCC tissues. Total RNA was resuspended in DEPC water, cleaned up with a Qiagen RNeasy column, and quantified by UV-Vis spectroscopy. The RNA was then qualified on an Agilent 2100 Bioanalyzer. One ug of total RNA from control and experimental animals was separately amplified and labeled with either Cy3- or Cy5-labeled CTP (Perkin Elmer, Wellesley, MA, USA) with an Agilent low input linear amplification kit (Agilent Technologies, Palo Alto, CA, USA) according to the manufacturer’s protocol. After labeling and cleanup, amplified RNA was quantified by UV-vis spectroscopy. Samples (0.75 ug each) of Cy3- and Cy5-labeled targets were combined and hybridized to an Agilent catalog 44K feature mouse oligo array for 17 h at 60°C. In each assay, a hepatic RNA sample from an experimental animal was hybridized against a pooled RNA sample composed of equal amounts of RNA from the livers of five unmanipulated 7-month-old control transgenic liver mixture (Tg mix). After hybridization, arrays were washed consecutively in solutions of 6 x SSPE with 0.005% *N*-lauroylsarcosine and 0.06 x SSPE with 0.005% *N*-lauroylsarcosine for 1 min each at room temperature. This was followed by a 30 s wash in Agilent stabilization and drying solution.

### Microarray data analysis

Data were extracted from the resulting images using Agilent's Feature Extraction Software (Agilent Technologies). Expression analysis for all replicate microarray experiments was performed with GeneSpring GX, where its expression ratio (experiment group = Cy5 / control group = Cy3) was calculated and converted using the base two logarithm. If flags ("gIsPosAndSignif", "rIsPosAndSignif", "gIsWellAboveBG", or "rIsWellAboveBG"), indicating as reliable fluorescence probes, were 0, those probes were eliminated from the obtained data as well as probes with 1 or -1 in ControlType. For the logarithm ratio of each chronological sample in the unmanipulated control Tg mix, two color microarray analysis was performed by setting the samples with log2 ratios of 0. False Discovery Rate (FDR) with Welch *P*-values and RankProd FDR were respectively corrected for multiple testing with Benjamin-Hochberg (BH) procedure and RankProduct. While Welch *P*-values were calculated from TM4 MeV_4_6_1, R was used for RankProduct FDR.

To observe assembled signaling pathways with differentially expressed genes, gene set enrichment analysis (GSEA) was performed for listing 259 pathways in Pathway Studio ver. 8 in a pathway analysis software. The comparison combinations used in this analysis were the average values of log-ratios against Tg mix of Tg7d, Tg1M, Tg3M, Tg6M, Tg9M, Tg12M, Tg15M, Tg18M, and tumor (Tg18M) and the log ratios of Tg18M/Tg mix and tumor (Tg18M)/ Tg mix. A heatmap was generated by TM4 MeV based on average log2 ratios from each group of genes, constructing pathways with *P*-values of less than 0.01 from comparisons of either Tg 18M vs Control or tumor (Tg18M) vs Tg18M on the GSEA analysis. The average linkage method, based on Pearson correlation coefficient, was used for gene hierarchical clustering. If a gene corresponded to multiple probes, the probe with the highest dispersion among the 45 samples was selected as the representative probe, and its expression value was used. All datasets have been deposited at National Center for Biotechnology Information/Gene Expression Omnibus under accession number GSE103205.

### Real-time PCR analysis

Quantitative gene expression levels were determined using real-time PCR with the ABI Prism 7900HT Sequence Detection System (Applied Biosystems, Foster City, CA) and SYBR Green I dye or TaqMan MGB probes (FAM^TM^ dye labeled). Primers were created using Applied Biosystems Primer Express Software version 2.0 or purchased by Applied Biosystems Assays-on-Demand Gene expression products. For amplification diluted cDNA was combined with a reaction mixture containing SYBR Green PCR core reagents (Applied Biosystems, catalog no. 4304886) or with TaqMan universal PCR Master Mix (Applied Biosystems, catalog no. 4304437) according to the manufacturer's instructions. The ß-actin gene was used as the endogenous control for normalizing initial RNA levels.

### Immunohistochemical analysis

The liver tissues were fixed in buffered zinc formalin, embedded in paraffin, and sectioned. The localization of 8-nitroguanine (8-NG) and inducible nitric oxide synthase (iNOS) was assessed by double immunofluorescence labeling.[17] The paraffin sections were incubated with the anti-8-nitroguanine antibody (1 μg/mL) and anti-iNOS (1:300; Sigma, St Louis, MO, USA) overnight at room temperature. To confirm 8-nitroguanine formation in genomic DNA, we pretreated the indicated tissue sections with 10 μg/mL RNase at 37°C for 1 h as previously described.[18] Sections were then incubated for 3 h with an Alexa 594-labeled goat anti-rabbit IgG antibody and an Alexa 488-labeled goat anti-mouse IgG antibody (both 1:400; Molecular Probes, Eugene, OR, USA). Stained sections were examined using an inverted Laser Scan Microscope (LSM 410; Zeiss, Gottingen, Germany). For the expression of proliferating cell nuclear antigen (PCNA) and signal transducer and activator of transcription 3 (STAT3), immunohistochemistry was performed using an anti-PCNA monoclonal primary antibody (PC10; DAKO, Carpinteria, CA) and an anti-STAT3 monoclonal primary antibody (79D7; Cell Signaling, Danvers, MA), respectively, and Envision+ kits according to the manufacturer’s instructions (DAKO).

### Western blotting

Liver samples were prepared using radio-immunoprecipitation assay (RIPA) lysis buffer as previously described.[19] Anti-STAT3 monoclonal (79D7; Cell Signaling), anti-mouse p53 polyclonal (Leica, Newcastle, UK), anti-phospho-p53 (Ser6 and Ser15) (Cell Signaling) and anti-β-actin monoclonal (Sigma–Aldrich, St. Louis, MO) antibodies were used for protein detection. Immune complexes were visualized using enhanced chemiluminescence detection reagents (Amersham Biosciences, Piscataway, NJ) in accordance with the manufacturer’s protocol.

## Results

### Immunohistochemical changes of hepatocytes in HBV transgenic mice

**Fig 1:**
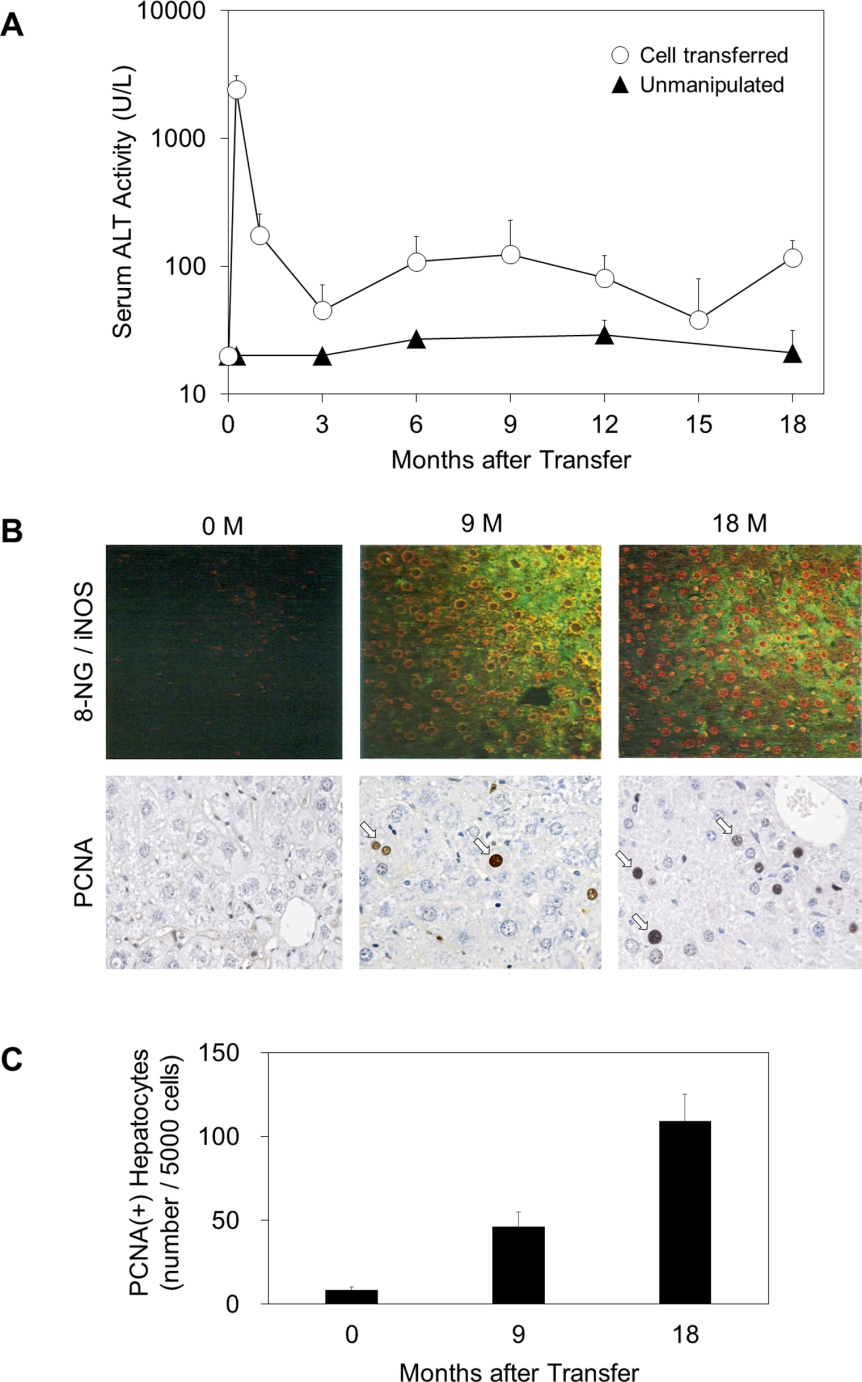
Course of liver disease in a transgenic mouse model of chronic hepatitis B. (A) Kinetics of serum alanine aminotransferase (ALT) in HBV transgenic mice (open circles; n = 40) were monitored after transferring spleen cells from syngeneic nontransgenic donors that had been previously immunized with recombinant vaccinia virus HBs-vac. All results were compared with unmanipulated age- and sex-matched transgenic mice (filled triangles; n = 20). Adoptive transfer of splenocytes was performed on day 0. Serum ALT activity was monitored to evaluate liver injury. Results were expressed as mean units per liter ± S.D. of ALT activity. (B) Inflammation-induced, reactive nitrogen species and regenerative proliferation of hepatocytes were estimated immunohistochemically using a double immunofluorescence technique for 8-nitroguanine (8-NG) (nuclear, red) and inducible nitric oxide synthase (iNOS) (cytoplasmic, green) expression and anti-proliferating cell nuclear antigen (PCNA) solution at 0 (untreated), 9 and 18 months. PCNA(+) hepatocyte nuclei are indicated with arrows. Original magnifications: x 100 (8-NG / iNOS) and x 400 (PCNA). (C) Quantitative morphometric analysis of PCNA^+^ hepatocytes. Fifty high power (x 400) fields representing 2 mm^2^ of liver tissue were examined. Results are expressed means ± S.E. per 1,000 hepatocytes. Each group represents three animals.

After the immunological reconstitution of HBV transgenic mice with the virus-specific CD8^+^ CTLs, serum ALT activity increased from preinjection levels of 20–40 U/L to peak levels (mean + SD [U/L]: 2,411 + 668) within 7 d, fell progressively thereafter, and remained at least two to three times above normal until 18 months at which time all mice developed multiple liver tumors (Fig 1A). In contrast, the unmanipulated HBV transgenic controls showed no serum ALT elevation, indicating no liver disease for the entire experimental period, as previously observed.[10] In addition, we have previously observed that sham-treated HBV transgenic mice which had been thymectomized, irradiated, and reconstituted with immunologically tolerant transgenic donor bone marrow cells and splenocytes developed no liver disease or no liver tumors.[10, 11]

To evaluate the inflammation-induced, reactive nitrogen species and regenerative proliferation of hepatocytes, the liver specimens were examined immunohistochemically using a double immunofluorescence technique for 8-NG (nuclear, red) and iNOS (cytoplasmic, green) expression and anti-PCNA solution at 0 (untreated), 9 and 18 months (Fig 1B). Cytoplasmic iNOS expression was observed at 9 months and nuclear 8-NG expression was remarkably enhanced in nontumor liver tissues at 18 months. In addition, the number of PCNA^+^ hepatocytes was greatly increased at 9 months and further elevation was seen at 18 months (Fig 1C). And, we have shown that TUNEL^+^ hepatocellular apoptosis was induced in this model, especially in the early phase.[11, 12] The results collectively demonstrated that the long-term HBV-specific immune responses caused the nitrative DNA lesions and the high regenerative proliferation of hepatocytes in the transgenic mice.

### Serial changes of gene expression profile in the liver of HBV transgenic mice

To investigate the serial changes of gene expression profiles in the mouse livers, five immunologically reconstituted HBV transgenic mice were sacrificed on day 7 and every three months until 18 months. The comprehensive gene expression profiles of liver specimens were analyzed by microarray (S1 Table). For the logarithm ratio of each chronological sample in the unmanipulated control Tg mix, two color microarray analysis was performed by setting the samples with log2 ratios of 0. To observe assembled signal pathways with differentially expressed genes, GSEA was performed for listing 259 pathways in a pathway analysis software. We focused on the most significant sixteen pathways (*P* < 0.05) at 18 months in nontumor liver specimens because they may represent the pathways with high procarcinogenic potentials as the background of HCC (Table 1).

**Table 1:** Serial changes of signaling pathways in a model of chronic Hepatitis B.

	7 d			1 M			3 M			6 M			9 M			12 M			15 M			18 M			18 M—Tumor		
Signaling Pathways*	Norm.ES	M.change	p-value	Norm.ES	M.change	p-value	Norm.ES	M.change	p-value	Norm.ES	M.change	p-value	Norm.ES	M.change	p-value	Norm.ES	M.change	p-value	Norm.ES	M.change	p-value	Norm.ES	M.change	p-value	Norm.ES	M.change	p-value
CCR2/5 -> STAT signaling	1.933	4.377	< 0.003	1.939	1.545	< 0.003	1.334	1.222	0.107	1.840	1.306	< 0.003	1.659	1.186	0.021	1.841	1.602	0.003	1.886	1.190	0.004	1.834	1.622	< 0.003	1.738	1.491	< 0.003
CCR5 -> TP53 signaling	1.917	3.784	< 0.003	1.924	1.545	< 0.003	1.580	1.097	0.018	1.755	1.230	< 0.003	1.744	1.160	< 0.003	1.726	1.594	0.007	1.666	1.149	0.013	1.677	1.495	< 0.003	1.508	1.226	0.040
NeurotensinR -> ELK-SRF/AP-1/EGR signaling	0.776	-1.008	0.760	-1.038	1.070	0.380	-1.230	-1.023	0.156	-1.488	-1.046	0.053	1.007	-1.005	0.462	-1.709	1.043	< 0.003	-1.077	-1.013	0.313	-1.751	-1.061	< 0.003	-0.993	-1.042	0.442
T-cell receptor -> NF-kB signaling	1.944	1.856	< 0.003	1.603	1.282	0.006	1.061	1.051	0.395	1.713	1.158	< 0.003	0.962	1.037	0.511	1.433	1.151	0.057	1.684	1.176	0.008	1.605	1.307	0.003	1.636	1.310	0.013
MacrophageR -> CEBPB/NF-kB signaling	1.694	1.309	< 0.003	1.445	1.077	0.057	0.582	1.033	0.933	0.940	1.123	0.539	1.094	1.054	0.376	1.389	1.221	0.108	1.476	1.211	0.043	1.672	1.191	0.003	1.190	1.249	0.255
CCR1 -> STAT signaling	1.758	1.988	0.004	1.765	1.545	< 0.003	1.533	1.094	0.026	1.736	1.167	< 0.003	1.868	1.360	< 0.003	1.674	1.594	0.007	1.925	1.610	< 0.003	1.754	1.495	0.004	1.708	1.618	0.005
T-cell receptor -> CREBBP signaling	2.029	1.948	< 0.003	1.535	1.282	0.023	1.152	1.043	0.272	1.446	1.102	0.059	0.627	1.054	0.949	1.353	1.124	0.075	1.617	1.133	0.012	1.630	1.307	0.009	1.230	1.197	0.181
CD2 -> NFATC1 signaling	1.677	1.500	< 0.003	1.447	1.384	0.054	1.441	1.203	0.068	1.747	1.200	< 0.003	0.640	1.052	0.919	1.443	1.124	0.060	1.373	1.176	0.100	1.559	1.384	0.011	1.071	1.198	0.350
T-cell receptor -> NFATC signaling	2.001	1.504	< 0.003	1.558	1.282	0.012	1.146	1.042	0.285	1.620	1.064	0.016	0.452	1.035	0.996	1.462	1.124	0.041	1.422	1.106	0.087	1.552	1.307	0.012	1.214	1.198	0.173
IL1R -> STAT3 signaling	1.514	1.468	0.040	1.124	1.145	0.337	0.927	1.060	0.559	1.501	1.010	0.030	1.484	1.017	0.053	1.430	1.224	0.068	1.934	1.233	< 0.003	1.573	1.241	0.021	1.345	1.102	0.127
TLR1/2/6 -> NF-kB signaling	1.542	1.309	0.025	1.023	1.123	0.448	1.106	1.120	0.365	1.533	1.071	0.023	1.332	1.061	0.139	1.270	1.234	0.177	1.648	1.172	0.019	1.505	1.331	0.028	1.758	1.215	0.009
TLR4 -> IRF signaling	1.467	1.320	0.045	1.561	1.145	0.010	1.338	1.140	0.138	1.317	1.110	0.159	1.502	1.017	0.066	1.533	1.224	0.043	1.315	1.110	0.119	1.527	1.108	0.030	1.360	1.156	0.119
EctodysplasinR -> NF-kB signaling	0.900	1.120	0.603	1.109	1.101	0.340	1.035	1.154	0.420	1.452	1.260	0.101	1.667	1.236	0.012	1.473	1.376	0.029	1.357	1.219	0.128	1.543	1.540	0.031	1.276	1.304	0.164
EphrinR -> STAT signaling	1.277	1.420	0.179	1.184	1.046	0.265	1.239	1.186	0.201	1.403	1.133	0.080	1.126	1.084	0.307	1.311	1.094	0.154	1.659	1.154	0.022	1.524	1.215	0.035	1.485	1.360	0.039
IL7R -> FOXO/NF-kB signaling	1.414	1.309	0.088	0.978	1.193	0.507	1.301	1.120	0.141	1.499	1.205	0.044	1.356	1.124	0.111	1.217	1.253	0.248	1.446	1.176	0.084	1.492	1.339	0.036	1.213	1.167	0.254
InsulinR -> ELK-SRF/SREBF signaling	-1.228	-1.008	0.160	-0.635	1.070	0.909	-1.099	-1.053	0.341	-1.491	-1.048	0.079	-1.000	1.003	0.459	-1.959	1.016	0.010	-1.138	-1.040	0.286	-1.578	-1.047	0.037	-1.260	-1.042	0.166

*Signaling pathways were listed based on significant p-values at 18 M.

Abbreviations: Norm.ES, Normalized ES; M.change, Median change.

**Fig 2:**
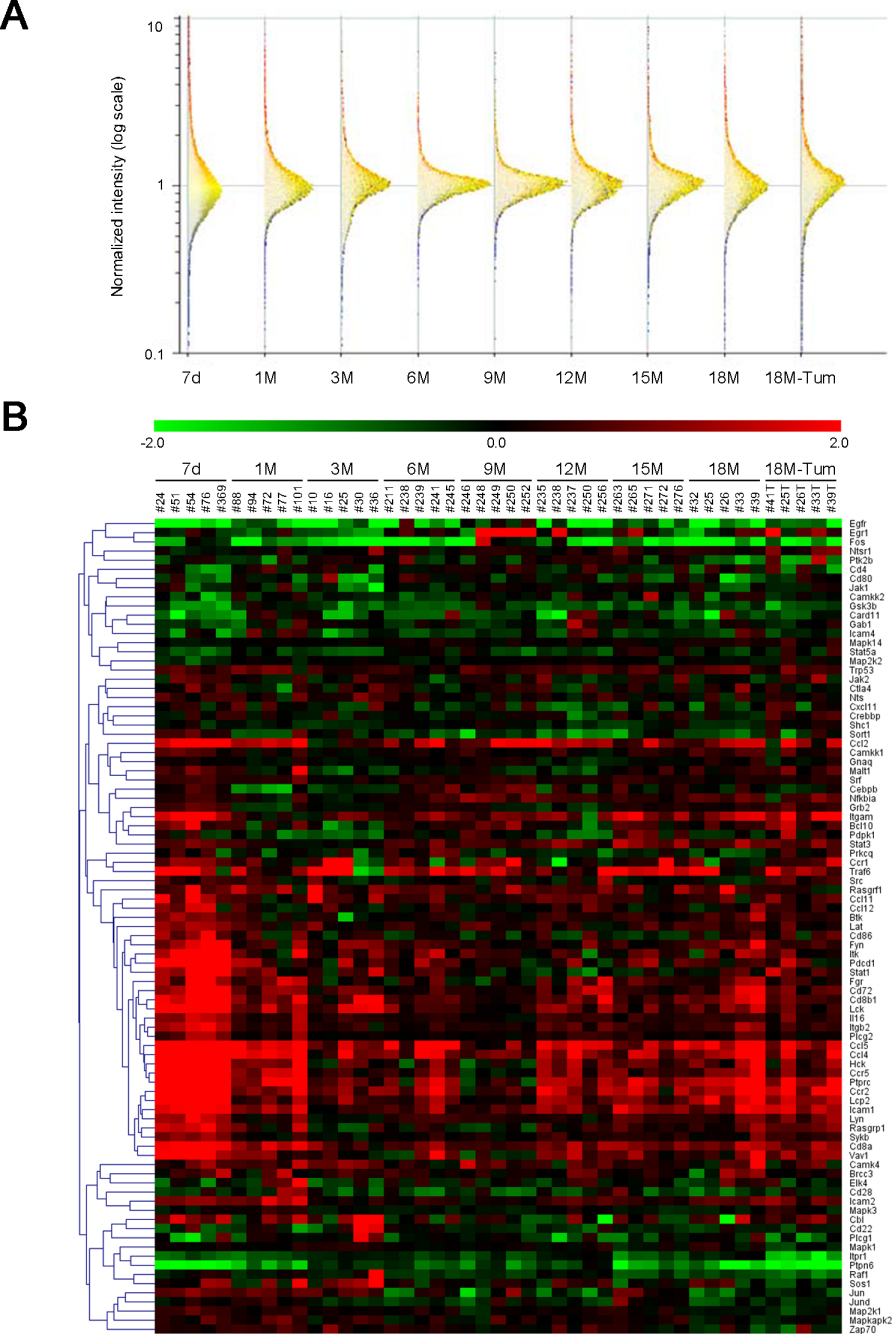
Gene expression profiles in the model of chronic hepatitis B. (A) Total RNA isolated from liver tissues were analyzed by microarray. For the logarithm ratio of each chronological sample in unmanipulated control Tg mix, two color microarray analysis was performed by setting the samples with log2 ratios of 0. Gene expression data at each time point were well normalized by a robust multiarray analysis (RMA) program. (B) A heatmap was generated by TM4 MeV based on average log2 ratios from a group of genes, constructing the significant pathways with *P*-values of less than 0.01 from comparisons of Tg 18M vs control Tg mix on the gene set enrichment analysis (GSEA) in Table 1. Each cell in the matrix represents the expression of a gene in an individual sample. Red and green cells depict high and low expression levels, respectively, as indicated by the scale bar (fold change in expression).

When the levels of selected sixteen pathways were monitored for 18 months, three chemokine-related pathways (CCR2/5 -> STAT, CCR5 -> TP53 and CCR1 -> STAT) consisting of CCL2, CCL3L1, CCL4, CCL5, CCL7, CCL11, CCL13, CXCL11, CCR1, CCR2, CCR5, JAK1, JAK2, STAT1, STAT3, STAT5A, MAPK14 and TP53 (p53) molecules were constitutively activated for the whole process of chronic liver disease. In addition, three T-cell receptor (TCR)-related pathways (TCR -> NF-kB, TCR -> CREBBP and TCR -> NFATC) consisting of CD4, CD8A, CD8B, CD22, CD28, CD72, CD80, CD86, NFKBIA, VAV1, LYN, PTPRC, SYK, PLCG1, PRKCQ, FYN, BCL10, ITK, PTPN6, CARD11, CTLA4, IL16, MAP3K7, LAT, PDCD1, LCK, BTK, ZAP70, MALT1, TRAF6, LCP2, PLCG2, ITPR1, CAMK4, CREBBP, CAMKK2, CAMKK1, NFAT5, NFATC4, NFATC3, NFATC1 and NFATC2 molecules were highly significant at both early (7d and 1M) and late (15M and 18M) time points. Additionally, a heatmap was generated based on average log2 ratios from each group of genes, constructing pathways with *P*-values of less than 0.01 from comparisons of Tg 18M vs control Tg mix on the GSEA analysis (Fig 2). Indicated many molecules were activated at both early and late points. The levels of gene expression were confirmed using RT-PCR (not shown).

### STAT3 and p53 protein expression

**Fig 3:**
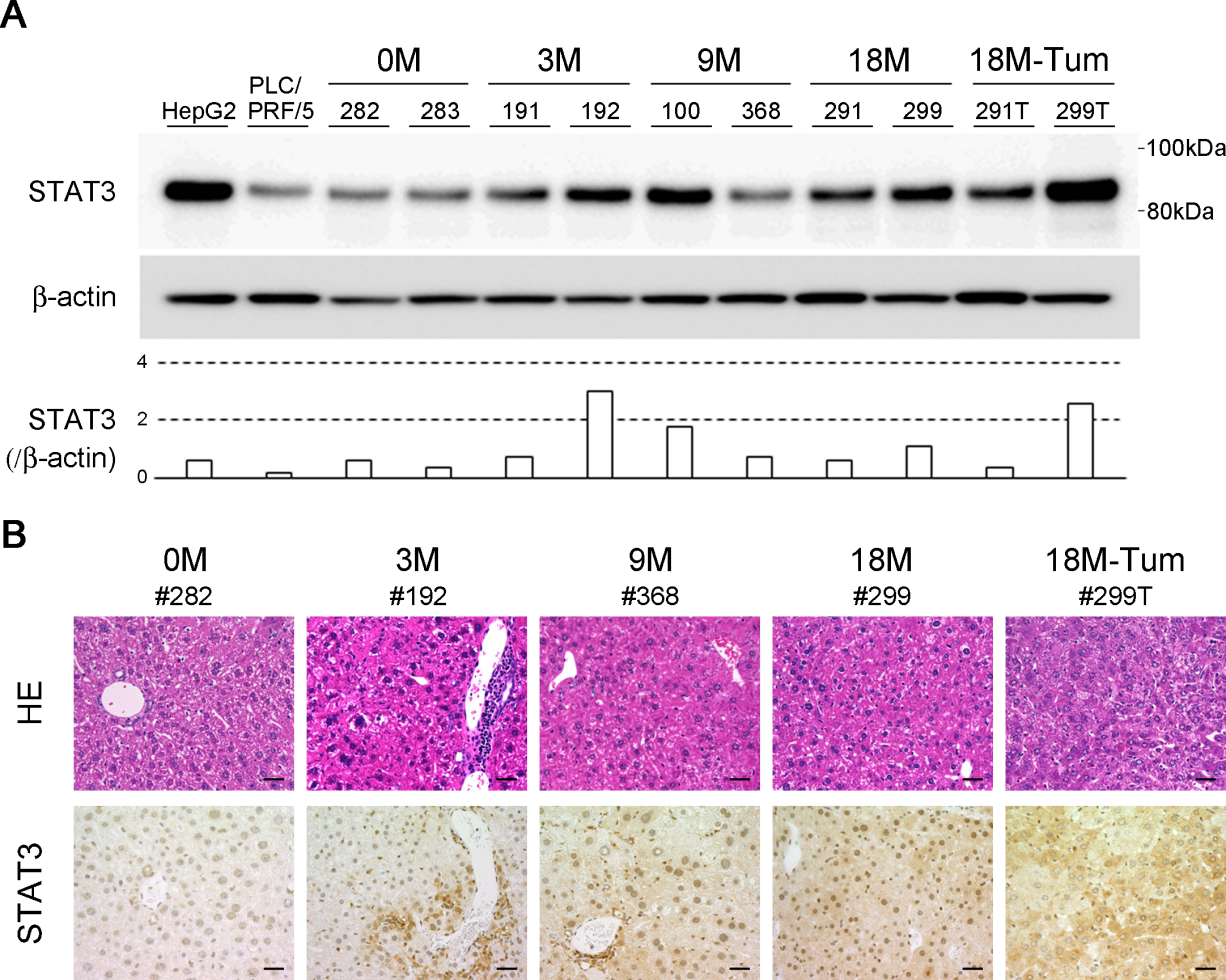
STAT3 expression in the model of chronic hepatitis B. (A) STAT3 levels in the liver tissues at the indicated time points (two representatives of each), the tumor tissues (18M-Tum) and HCC cell lines (HepG2 and PLC/PRF/5) were evaluated by Western blotting, and normalized to β-actin. (B) The liver tissues were stained with hematoxylin and eosin, and analyzed immunohistochemically. #282 and #283 mice (0M: unmanipuladed) were 24-month-old. Original magnifications, x 200 (bar represents 50 μm).

**Fig 4:**
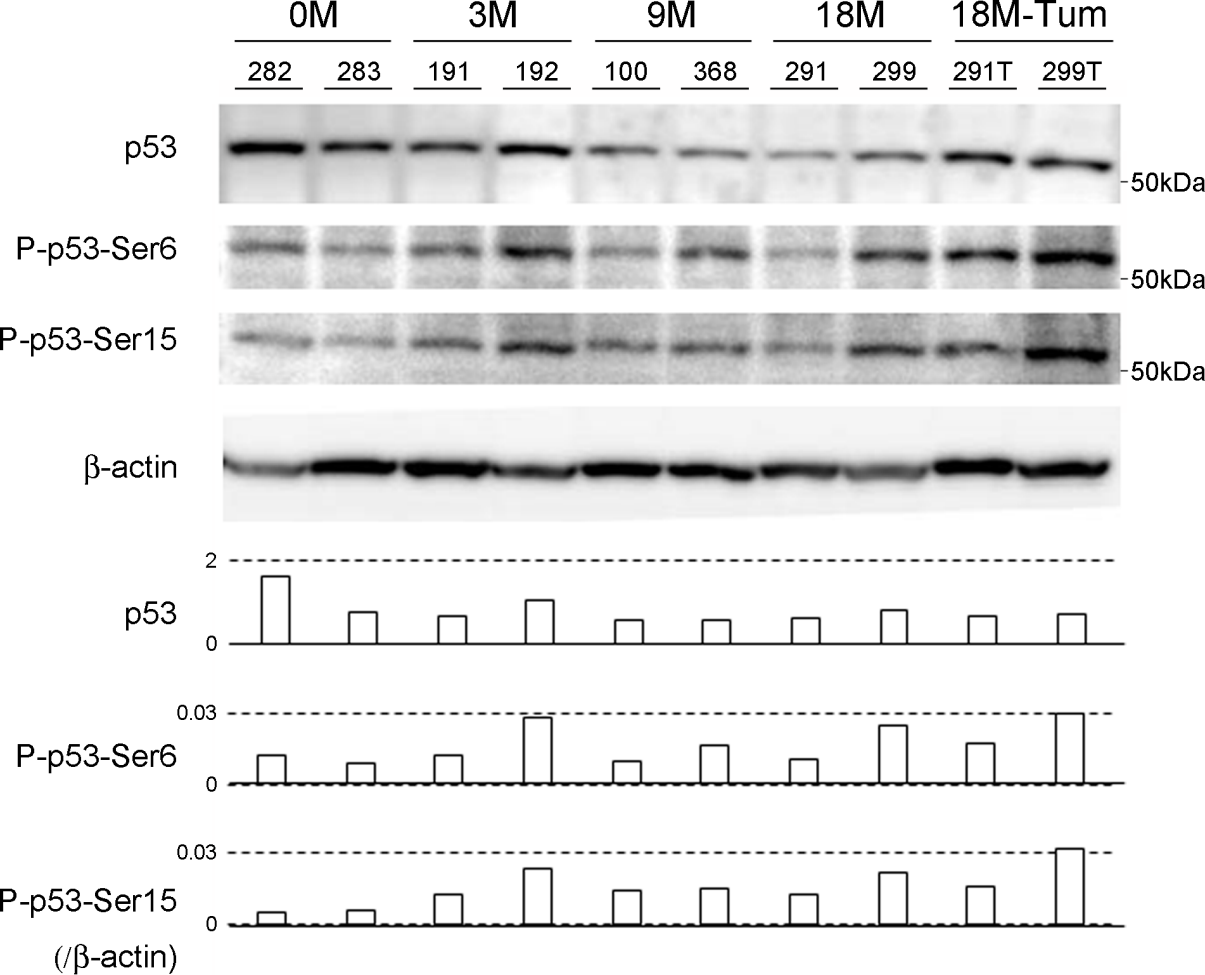
TP53 expression in the model of chronic hepatitis B. Total TP53 levels and phosphorylated TP53 at serines 6 and 15 (p-p53-Ser 6 and -Ser 15) in the liver tissues at the indicated time points (two representatives of each) and the tumor tissues (18M-Tum) were evaluated by Western blotting, and normalized to β -actin.

Among the molecules shown to be activated in the microarray analysis, the protein levels of STAT3 and p53 in liver tissues were assessed by Western blot and immunohistochemistry. Compared to the unmanipulated HBV transgenic controls (#.282 and #.283), the mice with chronic hepatitis expressed higher levels of STAT3 protein in a Western blot analysis (Fig 3A). Interestingly, STAT3 protein was expressed in infiltrating immune cells at 3 months and in part of hepatocyte nuclei at 9 months. The expression of STAT3 was greatly enhanced in all hepatocyte nuclei at 18 months and in the cytoplasm of HCC tissue (Fig 3B). Although the amount of TP53 was virtually constant during liver disease, the levels of phosphorylated TP53 at serines 6 and 15 (p-p53-Ser 6 and -Ser 15) were increased in diseased liver tissues (Fig 4). In addition, upon comparing the gene expression profile of tumor tissue with that of background nontumor liver (S1 Table), the EGF- and VEGF-mediated pathways were distinctively elevated in HCC (Table 2). The levels of gene expression were confirmed using RT-PCR (not shown). Taken together, in the gene expression profile analysis, TCR- and chemokine-mediated pathways were enhanced in the process of chronic hepatitis, and, subsequently, EGF- and VEGF-mediated pathways were induced in the development of HCC.

**Table 2:** Tumor-specific changes of signaling pathways.

	18M-Tumor / 18M (Nontumor)		
Signaling Pathways*	Norm.ES	M.change	p-value
EGFR/ERBB2 -> CTNNB signaling	1.925	1.003	< 0.003
EGFR/ERBB -> STAT signaling	2.089	1.021	< 0.003
EGFR -> CTNND signaling	1.940	1.239	< 0.003
VEGFR -> STAT signaling	1.805	1.215	< 0.003
CD2 -> NFATC1 signaling	-1.719	-1.300	< 0.003
ThrombinR -> STAT1 signaling	1.917	1.356	< 0.003
IFNGR -> STAT signaling	1.824	1.214	0.006
IL22R -> STAT3 signaling	1.693	1.068	0.007
TLR1/2/6 -> NF-kB signaling	1.657	1.072	0.015
EGFR -> NCOR2 signaling	1.483	1.050	0.018
TNFRSF1A -> STAT signaling	1.638	1.215	0.024
IGF1R -> STAT signaling	1.607	1.068	0.025
ThrombopoietinR -> STAT signaling	1.680	1.227	0.026
SomatostatinR -> ATF1/TP53 signaling	-1.550	-1.044	0.029
EGFR -> ZNF259 signaling	1.712	-1.073	0.034
IL5R -> STAT signaling	1.525	1.068	0.040
CCR5 -> TP53 signaling	-1.453	-1.020	0.047
TLR -> AP-1 signaling	1.600	1.005	0.048
VEGFR -> CTNND signaling	1.633	1.239	0.048

*Signaling pathways were listed based on significant p-values in 18M-Tumor / 18M (Nontumor).

Abbreviations: Norm.ES, Normalized ES; M.change, Median change.

## Discussion

The current study found that HBV-specific immune responses caused nitrative DNA lesions and the high regenerative proliferation of hepatocytes, activated the chemokine- and TCR-mediated intracellular pathways in long-term persistent hepatitis, and subsequently induced EGF- and VEGF-mediated procarcinogenic processes. Among the molecules investigated, the protein levels of STAT3 were greatly enhanced in all hepatocyte nuclei and further in the cytoplasm in HCC tissue, and the levels of phosphorylated TP53 (p-p53-Ser 6 and -Ser 15) were increased in liver tissues. The results suggested serial changes of gene expression profiles in chronic liver diseases that eventually terminate in HCC development.

After the immunological reconstitution of HBV transgenic mice, the acute phase of liver injury was observed within one month and the chronic phase inflammation was seen thereafter. We have shown that HBV-specific CTL responses and intrahepatic cytokine profiles including IL-1β, IL-6 and IFN-γ are detectable in these transgenic mice 17 months after reconstitution.[10] In support of this notion, the chemokine- and TCR-mediated pathways were enhanced in the liver tissues of chronic inflammation. The immunopathogenesis of chronic hepatitis may differ from that of acute hepatitis in patients with HBV infection.[20, 21] Although acute hepatitis B is associated with functionally efficient, multispecific antiviral T-cell responses, chronic virus persistence was reported to be characterized by an exhaustion of HBV-specific T-cell responses due to the tolerogenic effect of the liver environment. Unexpectedly, most of the pathways detected in 18M specimens were also activated in liver tissues at the earlier time points, 7d, 1M, 6M and 12M. The results indicated that the common immune-mediated mechanism was persistently enhanced in this model. Importantly, even if it might partially reflect the immunopathogenesis of patients with chronic hepatitis B, the current mechanism may be responsible for the hepatocellular carcinogenesis in the complex multifactorial process of persistently infected patients.

The EGF- and VEGF-mediated pathways were upregulated in HCC tissues surrounded by a chronic inflammatory microenvironment. The EGF-mediated pathway was reported to be deregulated in HCC[22] and to be linked to the HBx-NF-κB pathway, a part of the inflammation-fibrosis-cancer axis of the liver, via a non-receptor tyrosine phosphatase SHP2. [23, 24] The VEGF-mediated pathway was observed to be enhanced in the tumor angiogenesis of HCC.[25, 26] Additionally, high VEGF plasma levels are correlated with poor survival of patients with HCC.[27] A multikinase inhibitor that suppresses the phosphorylation of VEGF receptor 2 and EGF receptor was suggested to be a potential chemotherapeutic agent for HCC.[28] Taken together, the EGF- and VEGF-pathways may be critically involved in the inflammation-induced procarcinogenic process in chronic hepatitis B.

Three chemokine-related pathways (CCR2/5 -> STAT, CCR5 -> TP53 and CCR1 -> STAT) were constitutively activated in the entire process of chronic inflammation and carcinogenesis in the liver. In the procarcinogenic processes of other malignancies, CCR5 was observed to activate the STAT3 signal pathways for cancer stem cells to participate in tumor angiogenesis and for tumor-associated macrophages to induce the phenotypic shift.[29, 30] The activation of STAT3 downregulates the tumor suppressor p53 in colorectal cancer,[31] and further interaction of the STAT3-mediated signaling with p53 has been suggested in prostate cancer.[32] As seen in this model, inflammation-induced oxidative/nitrative stress leads multisite phosphorylation of p53 including Ser15 that is a major focal point in the activation[33–36] and is known to be phosphorylated by the ATM and ATR protein kinases.[37] Since oncogenic mutations were reported to occur primarily in the p53 gene (31%) in HCC specimens,[38, 39] its regulation may contribute to reduce the oncogenic potentials of chronic persistent inflammation in the liver.

The precise mechanisms of hepatocarcinogenesis still remain unanswered in chronic viral hepatitis. Within the whole spectrum of carcinogenic processes including liver cell injury,[20, 40] proliferation[41–43] and altered gene expression,[44–46] the current study design that evaluated gene expression profiles during long-term inflammation may be insufficient to consider the disease hypothesis. However, it is most likely, based on the remarkable similarity of the disease features in human viral hepatitis and the animal model used in this study,[10, 12] that the HBV-specific immunological events essentially contributes to the pathogenesis of HCC. Understanding the involvement of immune-mediated mechanisms in the HCC development should be critical not only for elucidating the pathogenesis of liver cancer, but also for creating new cancer-preventive strategies for patients with chronic hepatitis.

## Supporting information

S1 Table Comprehensive gene expression profiles by microarray in a transgenic mouse model of chronic hepatitis B. All datasets have been deposited at National Center for Biotechnology Information/Gene Expression Omnibus under accession number GSE103205.
(XLS)

## Abbreviations

8-NG: 8-nitroguanine
ALT: alanine aminotransferase
CTL: cytotoxic T lymphocyte
FDR: false discovery rate
GSEA: gene set enrichment analysis
HBsAg: hepatitis B surface antigen
HBV: hepatitis B virus
HCC: hepatocellular carcinoma
iNOS: inducible nitric oxide synthase
TCR: T cell receptor
Tg: transgenic
PCNA: proliferating cell nuclear antigen
STAT3: signal transducer and activator of transcription 3.
